# Acute rejection after kidney transplantation promotes graft fibrosis with elevated adenosine level in rat

**DOI:** 10.1371/journal.pone.0180211

**Published:** 2017-06-26

**Authors:** Mingliang Li, Yingbo Dai, Jun Lei, Jin Tang, Yihong Zhou, Bing Xia, Yang Xia, Guangming Yin

**Affiliations:** 1 Department of Urology, The Third Xiangya Hospital of Central South University, Changsha, Hunan, China; 2 Department of Urology, The First People's Hospital of Xiangtan City, Xiangtan, China; 3 Department of Biochemistry and Molecular Biology, The University of Texas–Houston Medical School, Houston, Texas, United States of America; University of Toledo, UNITED STATES

## Abstract

**Aims:**

Chronic allograft nephropathy is a worldwide issue with the major feature of progressive allograft fibrosis, eventually ending with graft loss. Adenosine has been demonstrated to play an important role in process of fibrosis. Our study aimed to investigate the relationship between adenosine and fibrosis in renal allograft acute rejection in rat.

**Materials and methods:**

Wistar rats and SD rats were selected as experimental animals. Our study designed two groups. In the allograft transplantation group, kidneys of Wistar rats were orthotopically transplanted into SD rat recipients, the same species but not genetically identical, to induce acute rejection. Kidney transplantations of SD rats to SD rats which were genetically identical were served as the control. We established rat models and detected a series of indicators. All data were analyzed statistically. *P*<0.05 was considered statistically significant.

**Results:**

Compared with the control group, levels of adenosine increased significantly in the allograft transplantation group, in which acute rejection was induced (P<0.05). Progressive allograft fibrosis as well as collagen deposition were observed.

**Conclusions:**

These findings suggested that level of adenosine was upregulated in acute rejection after kidney allograft transplantation in rat. Acute rejection may promote renal allograft fibrosis via the adenosine signaling pathways.

## Introduction

Kidney transplantation is the most effective treatment of end-stage renal failure, however, incidence of chronic allograft nephropathy (CAN) remains high, which finally leads to renal allograft dysfunction. One of the major features seen in CAN is progressive allograft fibrosis. Hence, attention has been focused on pathogenesis of renal graft fibrosis to improve preventment and treatment of CAN.

Except ischemic hypoxia and reperfusion injury [1,2], acute renal allograft rejection is considered to be the most important factor for allograft fibrosis [3,4]. One of the best-known signaling molecules induced under hypoxic conditions is adenosine [5,6]. Dai et al. found that A2B adenosine receptor-mediated IL-6 induction underlies the pathogenesis of chronic renal disease [7]. Tang et al. reported that increased adenosine levels contribute to ischemic renal fibrosis in the unilateral ureteral obstruction model [8]. Besides, pharmacological activation of the adenosine A(2A) receptor was demonstrated to rev**e**rse fibrosis, and reduce macrophage infiltration and inflammatory activation in rat nephrotoxic nephritis [9].

Renal graft will definitely experience a process of ischemic hypoxia and inflammation of acute rejection. Adenosine is an important molecule usually dysregulated under hypoxic and inflammatory conditions. We hypothesized that acute rejection may promote graft fibrosis through regulation of adenosine. In this study, we found that adenosine increased significantly in renal acute rejection experimental group, and upregulated level of adenosine was closely correlated with graft fibrosis. These findings provided an initial experimental basis for further study of specific mechanisms of adenosine promoting renal allograft fibrosis and a potential therapeutic target for CAN.

## Materials and methods

This study was carried out in strict accordance with the recommendations in the Guide for the Care and Use of Laboratory Animals of the National Institutes of Health. The protocol was approved by the Committee on the Ethics of the The Third Xiangya Hospital, Central South University. All surgery was performed under pentobarbital sodium anesthesia, and all efforts were made to minimize suffering.

All rats were similar in weight, age, diet and living environment. SD rat donors and SD rat recipients which are genetically identical were selected as the control group. Only ischemic hypoxia and reperfusion injury but not acute rejection occurred in control group. Wistar donors and SD recipients which are the same species but not genetically identical were selected as the experimental group. Acute rejection as well as ischemic hypoxia and reperfusion injury occurred in experimental group.

In order to prevent infection during perioperative period, the rats were fed in sterile environment, food and water being disinfected. Injection of antibiotics postoperatively was implemented to prevent infection. The rats were observed twice one day. Vitality and wound healing conditions were used to assess animal health and well-being. In this research, humane endpoints were in place to euthanize sick animals. We got approval from Institutional Animal Care and Use Committee (IACUC) and equivalent ethics committee, and abided by applicable national and international guidelines. Approval was received prior to beginning research. All outcomes were consistent with theoretical results, so there were not any adverse outcomes in animals. Besides effective anesthesia, minimizing the size of surgical wounds was effective method to to minimize suffering.

### Kidney transplantation method

Orthotopic kidney transplantation in rat is used for the establishment of animal model [10].

#### Donor organ harvest

The donor rat was anaesthetized with an intraperitoneal injection of pentobarbital sodium (10mg/kg). A cross incision was made from the sternum to the pubis. The intestines and stomach were moved to expose the left kidney. The aorta and inferior vena cava (IVC) were dissected at their junctions with the left renal artery and vein by ligating and dividing a few lumbar branches. The left ureter was dissected from the bladder to renal hilum. After exposing the ureterovesical junction, a small, elliptical patch of bladder containing the left ureterovesical junction was excised and used for urinary reconstruction in the recipient. The aorta above renal artery, the IVC above the renal vein and the right renal artery and vein were ligated with a 9–0 silk suture to minimize bleeding. The aorta was ligated and inserted epidural catheter below 1.5 cm from the renal artery for perfusion. The IVC was ligated and excised below 1.5 cm from the renal vein. The graft was then perfused via epidural catheter slowly and evenly in situ with 8–10 ml of cold, heparinized (100 unit/ml) renal graft preservation solution for 45 seconds. The IVC from donor at its junction with the renal vein from recipient was divided for transplantation. The aorta was divided approximately 2 cm below the renal artery. The kidney and associated vessels were then completely freed, removed and stored in saline at 4°C for further preparation. The donor rat was euthanized by cervical dislocation under anesthesia.

#### Recipient operation

After anesthesia, abdomen was opened via midline incision, and the bowel was moved to the right abdomen, covered with moist gauze. Isolating the left renal artery, vein and ureter. Clamping the left renal vein with a microvascular clamp at its junction with the IVC. Ligating the left renal artery at its junction with the aorta. The renal artery, vein and the ureter were cut at the renal hilum and the kidney was removed. The donor kidney was placed in ice intra-abdominally in the left flank and kept moist with a cold saline gauze. An end-to-end anastomosis was performed between the donor IVC below the renal vein and the recipient renal vein. The infrarenal aorta was isolated and cross-clamped after ligating the lumbar branches. A 10–0 nylon suture was placed through the full thickness of aorta and retracted to make an elliptical arteriotomy by a single cut. The aorta was irrigated with heparinized saline to clear intraluminal blood or clots. An end-to-side anastomosis was performed between the donor aorta and recipient aorta using interrupted 10–0 nylon sutures. Removing the aortic and renal vein clamps and the kidney was reperfused. Gentle pressure was applied to the anastomotic site with a dry cotton swab for 15 seconds after reperfusion. A cystotomy was made in the dome of the bladder. Sutures of 9–0 nylon were placed to anastomose the donor’s small bladder patch with the recipient’s bladder dome. The abdomen was closed in two layers with continuous 5–0 sterile nonabsorbable suture.

#### Post-operative care of recipient

After the closure of body wall and skin, cefoperazone (100 mg/kg) was administered subcutaneously to prevent infection. For the first 24 hours postoperatively the rat was placed in 37℃ incubator until fully awake.

#### Recipient nephrectomy

Tissue specimens of the recipients were taken for testing 1 week, 2 weeks, 3 weeks and 5 weeks post transplantation respectively. The abdomen was opened through a midline incision. The intestine was covered with saline-soaked gauze and carefully retracted to the left side. The graft was removed after ligating the right ureter, renal vein and renal artery. The graft was divided into three parts, two of which were dispensed rapidly frozen in liquid nitrogen for real-time PCR and adenosine level and the third part was fixed in 4% paraformaldehyde for paraffin embedding, sectioning, staining and immunohistochemistry testing.

### Successful rat model selection criteria

1. Reperfusion of the graft is good after kidney transplantation;
2. The recipient survives until nephrectomy;
3. Graft has good blood supply and there is no blood flow interruption or no obvious graft necrosis.

### Measurement

#### Quantification of graft adenosine levels

Recipients were anesthetized, and the grafts were rapidly removed and frozen in liquid nitrogen. Adenine nucleosides were extracted from frozen kidneys using 0.4 N perchloric acid, and adenosine was separated and quantified using reverse-phase HPLC as described previously[11].

#### Histological analysis

Recipients were anesthetized, and the graft were isolated and pressure-infused with 4% paraformaldehyde in PBS and fixed overnight at 4°C. Fixed tissues were rinsed in PBS, dehydrated through graded ethanol washes, and embedded in paraffin. 5-μm sections were collected on slides and stained with haematoxylin and eosin (H&E), Masson’s trichrome and immunohistochemistry.

#### Morphometric analysis of the graft fibrosis in Masson's trichrome stained sections

Ten consecutive non-overlapping fields of graft stained with the Masson's trichrome were analyzed. The fibrotic areas stained in light blue were picked up on the digital images using a computerized densitometry (ImagePro Plus, version 6.0, Media Cybernetics Inc) coupled to a microscope equipped with a digital camera as described[12–13]. The percentage of the fibrotic area relative to the whole area of the field was calculated (percent fibrosis area). The average densities of ten areas per graft were calculated and the SEM was indicated and the number of grafts was 4–5 for each category.

#### Morphometric analysis of graft fibrosis in immunohistochemistry sections

Immunohistochemistry was used for analysis of α-SMA and TGF-β1, which are commonly used to assess the degree of renal fibrosis. The areas stained in brown were picked up on the digital images using a computerized densitometry (ImagePro Plus, version 6.0, Media Cybernetics Inc) coupled to a microscope equipped with a digital camera as described [12,5]. The percentage of the fibrotic area was calculated (percent fibrosis area). The average density of ten areas per kidney was calculated and the SEM was indicated and the number of grafts was 4–5 for each category.

#### Total RNA isolation and real-time RT-PCR analysis

Total RNA was isolated using TRIzol reagent. RNase-free DNase was used to eliminate genomic DNA contamination. Transcript levels were quantified using real-time quantitative RT-PCR. Cyber green was used for analysis of procollagen I (α1), TGF-β1 and GAPDH using the following primers: procollagen I (α1), forward, 5’-gagccagcagattgagaaca t-3’, 5’-tactctccgctcttccagtca-3’. TGF-β1, forward, 5’-tatgacaacatcagggtctgga -3’, 5’-atcttcacggcaacttcttctc -3’. GAPDH, forward, 5’- acagcaacagggtggtggac -3’, 5’- tttgagggtgcagcgaactt -3.

### Statistical analysis

All data were presented as mean±SD. Statistical analysis was performed by ANOVA using GraphPad prism 5 software. *P*<0.05 was considered statistically significant.

## Results

### Adenosine is significantly increased after acute rejection

**Fig 1:**
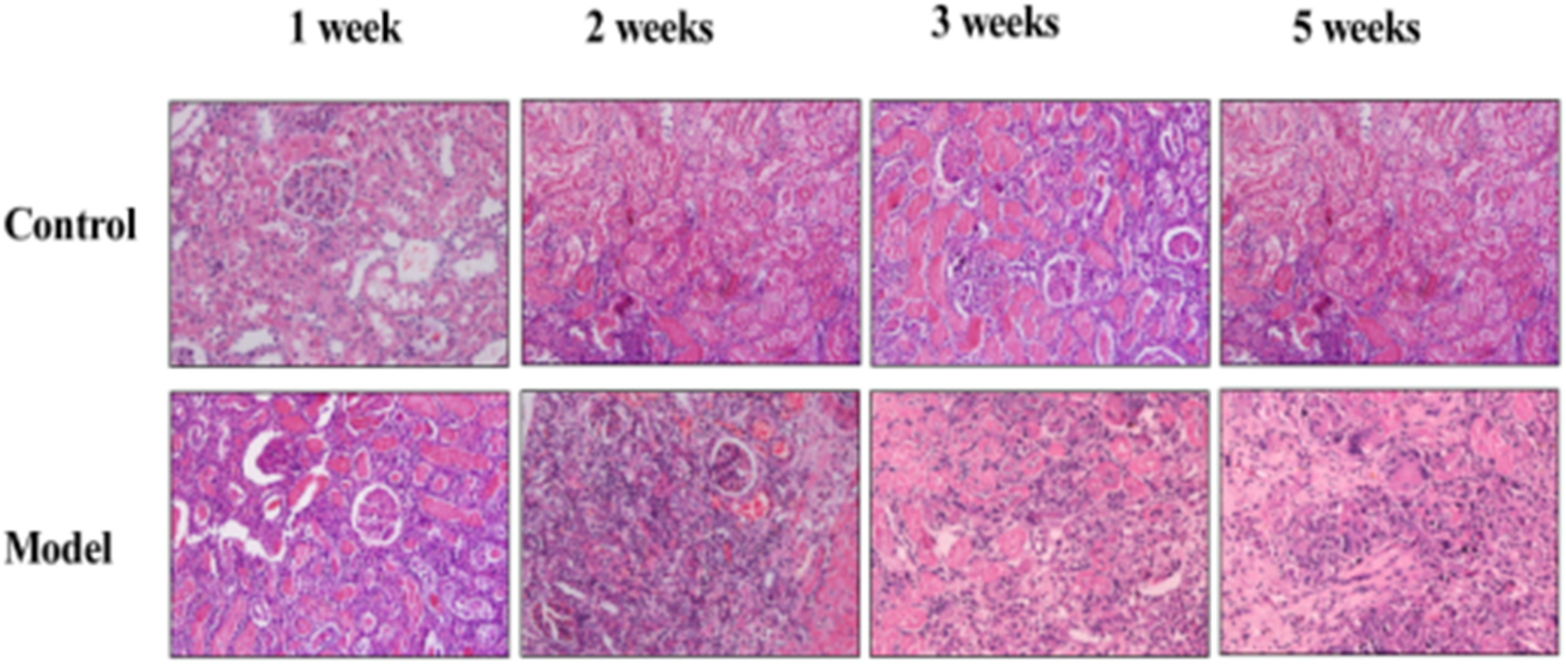
Histomorphological changes in two groups. H&E staining (200×). Acute rejection experimental group showed more inflammation infiltration and tissue damages than control group. Graft in experimental group have more inflammatory cell infiltration, tubular epithelial cell swelling and renal tissue damages.

**Fig 2:**
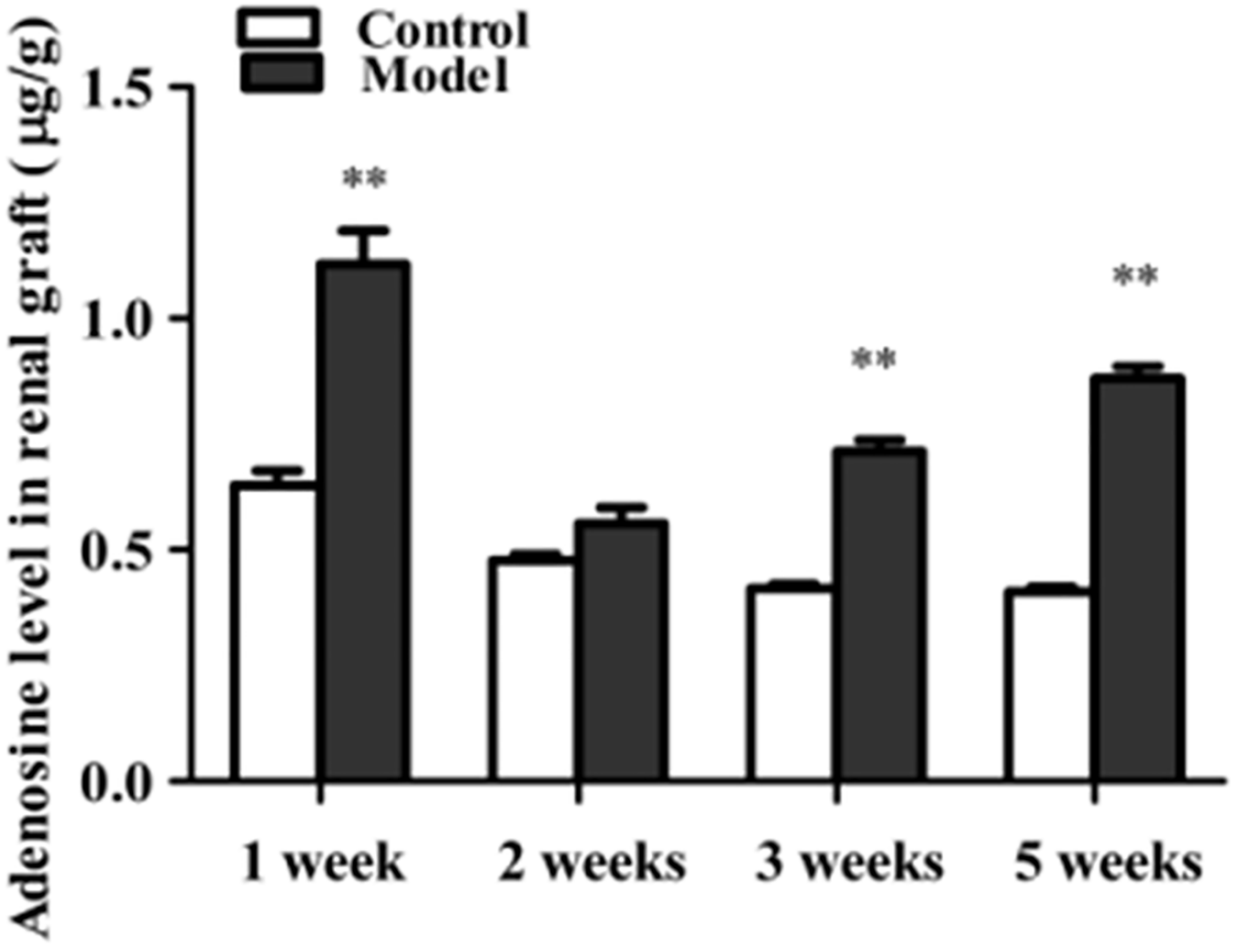
Comparison of changes in adenosine levels in two groups. Adenosine level was significantly upregulated in experimental group in which acute rejection existed. Adenosine levels in the kidney tissues were measured by HPLC. Data were described as mean ± SEM (n = 4 to 5). **P<0.01 versus control group.

To determine the significance of increased adenosine in allograft, in our study, Wistar rats’ kidneys were orthotopically transplanted to SD rat recipients, which are the same species with Wistar rat but not genetically identical (experimental group), to induce acute rejection. Kidney transplantations of SD rats to SD rats which were genetically identical were served as the control. In hematoxylin-eosin stained sections, acute rejection experimental group showed more extensively renal damagess and inflammation than control group in which only hypoxic-ischemic reperfusion injury but not acute rejection existed. Graft showed inflammatory cells infiltration, tubular epithelial cell swelling and renal tissue damagess (Fig 1). Compared with control group, the level of adenosine increased significantly in experimental group (Fig 2).

### Renal graft fibrosis is significantly accelerated in acute rejection experimental group

One of the major features of CAN is allograft fibrosis. To determine the correlation of between upregulated adenosine and renal graft fibrosis, histological examinations were conducted to characterize the renal fibrosis in each group.

**Fig 3:**
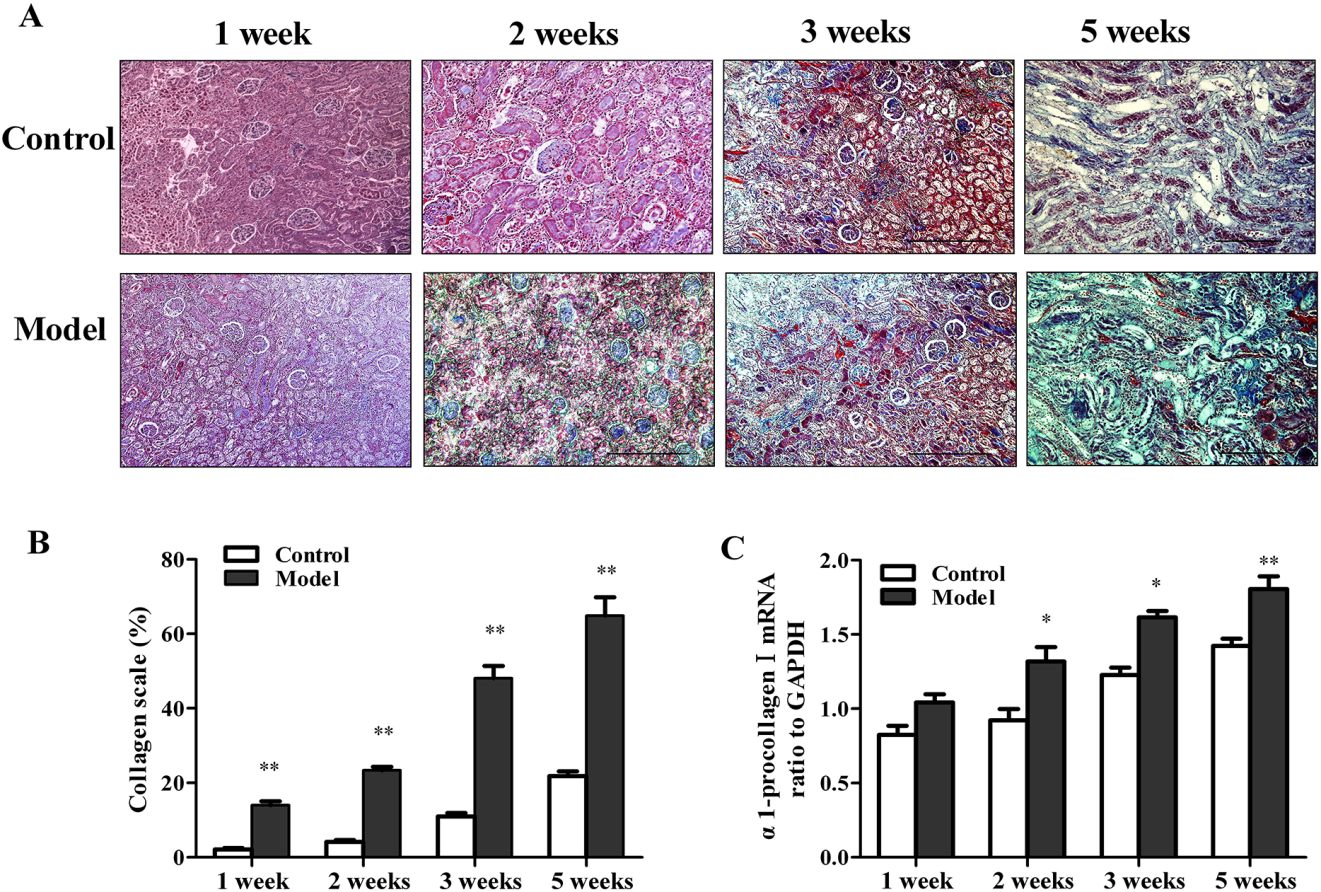
Comparison of collagen depositions in grafts. Renal graft fibrosis was significantly accelerated in experimental group. (A) Trichrome staining (100×). Graft in experimental group showed extensive fibrosis. (B) Quantitative image analyses showed collagen depositions in renal graft increased with time. (C) The fibrotic gene pro-collagen-mRNA expression was significantly increased in acute rejection experimental group. Data were expressed as mean ± SEM (n = 4 to 5). *P<0.05 versus control group. **P<0.01 versus control group.

Masson’s trichrome staining showed significant fibrosis in both glomeruli and interstitial areas between tubules. Compared with the control group, quantitative image analysis showed significantly increased collagen staining in renal graft in experimental group (Fig 3A and 3B).

**Fig 4:**
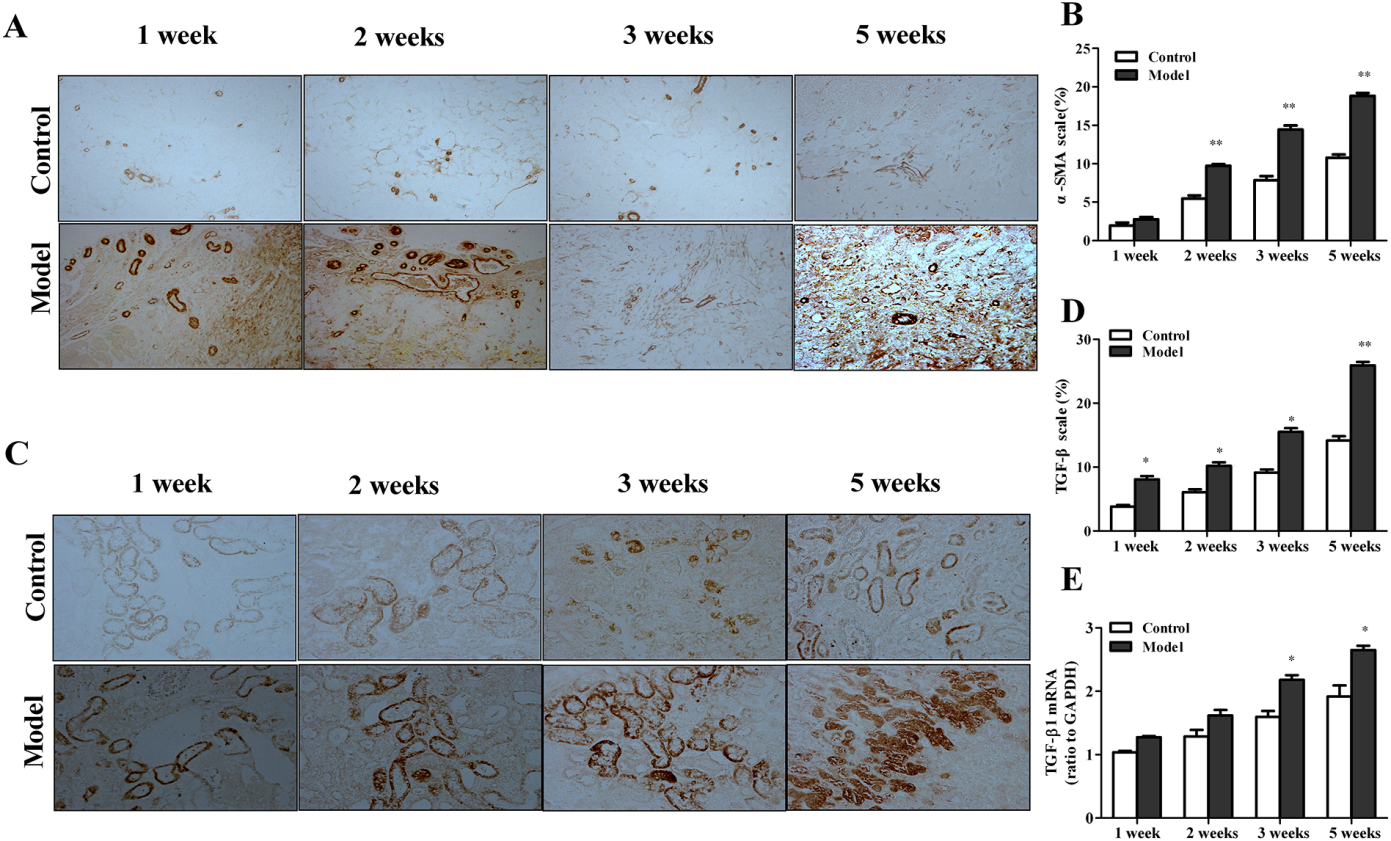
Comparison of specific molecules expressions in grafts. Renal graft fibrosis was significantly accelerated in acute rejection experimental group. (A) Immunohistochemistry staining (100×) showed that the fibrotic protein expression of α-SMA was significantly upregulated in experimental group. (B) Immunohistochemistry staining (200×) showed that the fibrotic protein expression of TGF-β1 in graft was significantly upregulated in experimental group. (C) Quantitative image analyses showed the protein expression of α-SMA was significantly upregulated in experimental group. (D) Quantitative image analyses showed the protein expression of TGF-β1 was significantly upregulated in experimental group. (E)The fibrotic gene TGF-β1 mRNA expression was significantly upregulated in experimental group. Data were expressed as mean±SEM (n = 4 to 5). *P<0.05 versus control group. **P<0.01 versus control group.

Furthermore, quantitative image analysis showed that the protein expression levels of α-SMA and TGF-β1 which are commonly used to assess the degree of renal fibrosis were significantly elevated in the grafts of the experimental group (Fig 4).

Consistent with histological studies, the mRNA levels of procollagen I (α1), TGF-β1 in grafts of the experimental group were significantly elevated showed by real-time PCR analysis (Fig 3C, Fig 4E).

To sum up, adenosine was demonstrated to be significantly upregulated in acute rejection condition after renal allograft transplantation. The degree of renal graft fibrosis was also significantly increased. These results suggest that acute rejection may promote renal graft fibrosis via upregulating adenosine in rat.

## Discussion

A role of adenosine (ADO) in graft fibrosis was initially demonstrated by the striking graft fibrosis and dysfunction observed in renal acute rejection models, which are similar CAN. These findings demonstrated that adenosine may be an important fibrotic signaling molecule promoting graft fibrosis.

Kidney transplantation is the best treatment for patients with end-stage renal failure. However, long-term effects of kidney transplant are not satisfactory, mainly due to chronic graft fibrosis, and gradually developed to CAN and graft disfunction. Acute rejection is considered one of the most important factors related to chronic renal graft fibrosis.

Adenosine has been studied as an immunomodulatory substance. Adenosine binds to adenosine receptors to produce anti-inflammatory effects. Adenosine can weaken the Th1 immune function in the inflammatory response by activating A_2A_R of the dendritic cell [13]. Besides, adenosine can inhibit proinflammatory cytokines (such as TNF-α, IL-6, IL-8, IL-12) [14] and positively regulate anti-inflammatory cytokines (e.g. IL-10) by activating A_2A_R on mononuclear cell membrane [15]. Adenosine also can stimulate bone marrow to enhance antiinflammatory effects by adenosine receptor pathway. ADO and purine nucleotide are upregulated when there is inflammation or injury in microenvironment, activating G protein-coupled receptor signaling pathways, increasing the number of immune cells which origin in bone marrow, and activating the regulation of immune to achieve the role of organ protecting and tissue repairing[15].

However, Adenosine is a double-edged sword. Persistently elevated adenosine also leads to excessive tissue repair, eventually resulting in organ or tissue fibrosis. Feoktistov found that elevated adenosine plays an important role in wound healing and tissue damagess by mediating adenosine receptor, A_2A_R. A_2B_R are involved in the formation of granulation tissue, and A_1_R may be involved in the formation of new blood vessels[16]. Similar mechanism is also found in the fibrosis of lung[17], liver[18] myocardial[19] and dermis[20]. Dai et al. found adenosine is one of predisposing factors contributing to penile fibrosis by mediated A_2B_R pathway[21,22].

This study designed model and control groups, aiming to study the correlation of acute rejection, adenosine and graft fibrosis. Adenosine was significantly increased in early postoperative period, and the level of adenosine of acute rejection group was higher than that of the control group, espeacieally 1 week postoperatively. Compared to the control group, the degree of inflammation in the experimental group was significantly higher showed by HE staining. Inflammation in the graft tissue mainly manifested large number of inflammatory cells infiltration, renal tubular edema or necrosis. As mentioned above, adenosine is an important immunomodulatory substance produced in condition of inflammation, ischemia and hypoxia. This suggests that increased adenosine may promote graft fibrosis in acute rejection, which is consistent with anti-inflammatory effect of adenosine in previous research.

Why does adenosine level significantly increased 1 week postoperatively? In early postoperative period, cytotoxicity and antibody-mediated inflammatory rejection lead to production of a large number of immune cells and activation of inflammatory cytokines, causing inflammation, tissue damages. In addition, vascular rejection causes necrotizing vasculitis of small arteries, leading to vascular injury, resulting in graft tissue ischemia and hypoxia, increasing renal tissue injury. Metabolism of graft tissue was accelerated in condition of inflammation, hypoxia and ischemia. ATP hydrolyzes into ADP, AMP and adenosine catalyzed by the enzyme of CD39 and CD73 step by step. Elevated adenosine has the function of producing anti-inflammatory effect, reducing tissue injury, regulating cell proliferation and tissue repair. In short, drastic increase of adenosine have a protective effect on graft.

2 weeks after transplantation, the experimental group still showed visible inflammatory cell infiltration, renal interstitial edema and tubular necrosis. However, renal graft injure was aggravated compared with that of 1 week after transplantation. Theoretically, adenosine should remain at a high level, playing a role in anti-inflammatory effects and tissue protection. However, adenosine level significantly decreased than 1 week after transplantation, which seemed to be contradicted with the previous theory. There may be three reasons for this situation. Firstly, adenosine is the product of cellular energy metabolism and adenosine level is related with the number of cells in tissue. In the condition of acute rejection containing severe cell injury and tissue necrosis in graft, the number of cells significantly decreased, so adenosine level reduced significantly 2 weeks after transplantation. Secondly, acute rejection may have the function of reducing cytokine, immune cells and inflammation, thereby adenosine level decreased. Thirdly, renal blood supply restored, ischemia-reperfusion injury and hypoxia reduced.

3 weeks after transplantation in experimental group, graft tissue showed more inflammatory cell infiltration, and renal interstitial and glomerular hyaline degeneration began to appear compared with control group. Graft fibrosis significantly increased 3 weeks postoperatively compared with 2 weeks postoperatively in experimental group. There was an upward trend of adenosine from 2 weeks postoperatively since persistent acute rejection, as well as increasing cell growth factor induced the fibrosis cells differentiation, proliferation and synthesis of extracellular matrix. Besides, the number of cells in graft increased. Adenosine, which is associated with the number of cells, increased and exerted anti-inflammatory effects. 5 weeks after transplantation, the trends and reasons were similar with that above.

In the control group, adenesine presented with a peak level in one week postoperatively, and then gradually decreased to normal level, while still higher than normal kidney tissue. Increased adenosine level is mainly caused by ischemia-reperfusion injury. Ischemic hypoxia and ischemia-reperfusion injury aroused, releasing large amounts of oxygen free radicals and inflammatory mediators, which resulted in increasing inflammatory response. Adenosine as an anti-inflammatory substances is often produced in conditions of ischemia and hypoxia conditions to protect the kidney tissue. It was found that isoflurane induced elevated levels of adenosine and reduced IRI, which were the same with our research results. The results confirmed ischemia-reperfusion injury is also an important factor leading to elevated adenosine.

After transplantation, graft experienced a pathological process from acute rejection inflammation, tissue damages to graft fibrosis. The pathological process in experimental group was significantly accelerated compared with control group. In this study, the main indicators of the evaluation of graft fibrosis were amount of collagen deposition, α-SMA protein expression, TGF-β1 gene and protein expressions and pro-collagen-gene expression. The degree of renal fibrosis and adenosine in experimental group significantly increased, confirming that acute rejection is an important factor in graft fibrosis. Increasing adenosine level early after kidney transplantation had a close relationship with graft fibrosis.

In conclusion, in renal allograft tissue experiencing acute rejection, adenosine is upregulated and allograft fibrosis is accelerated. The study found that acute rejection may be an important factor of elevated adenosine, which is highly correlated with graft fibrosis after kidney transplantation in rat. Acute rejection may promote renal allograft fibrosis via the adenosine signaling pathways.

## Supporting information

S1 File The original data of the histogram. Original data of adenosine levels in kidney, collagen scale, α1-collagen mRNA, α-SMA scale, TGF-β scale, TGF-β mRNA displayed in the histogram are listed in ditail in this file. The adenosine levels in the kidney of each rat in both control and model groups at different time postoperatively (1week, 2 weeks, 3 weeks, 5 weeks) are presented in Line 2–6 in the form and these data are statistically analyzed and shown in Fig 2. Similarly, Line 9–13 presents data for collagen scale of each rat in both control and model groups at different time postoperatively (1week, 2 weeks, 3 weeks, 5 weeks). Line 16–20 presents data for α1-collagen mRNA of each rat in both control and model groups at different time postoperatively (1week, 2 weeks, 3 weeks, 5 weeks). All the data listed in Line 9–20 are statistically analyzed and shown in Fig 3. Line 23–27 presents data for α-SMA scale of each rat in both control and model groups at different time postoperatively (1week, 2 weeks, 3 weeks, 5 weeks). Line 30–34 presents data for TGF-β scale of each rat in both control and model groups at different time postoperatively (1week, 2 weeks, 3 weeks, 5 weeks). Line 37–41 presents data for TGF-β mRNA of each rat in both control and model groups at different time postoperatively (1week, 2 weeks, 3 weeks, 5 weeks). All the data listed in Line 23–41 are statistically analyzed and shown in Fig 4.
(XLS)
